# Toward Personalized Web-Based Cognitive Rehabilitation for Patients With Ischemic Stroke: Elo Rating Approach

**DOI:** 10.2196/28090

**Published:** 2021-11-10

**Authors:** Alejandro Garcia-Rudolph, Eloy Opisso, Jose M Tormos, Vince Istvan Madai, Dietmar Frey, Helard Becerra, John D Kelleher, Montserrat Bernabeu Guitart, Jaume López

**Affiliations:** 1 Institut Guttmann Hospital de Neurorehabilitacio Badalona Spain; 2 Universitat Autònoma de Barcelona Barcelona Spain; 3 Fundació Institut d'Investigació en Ciències de la Salut Germans Trias i Pujol Badalona Spain; 4 Charité Lab for AI in Medicine Charité Universitätsmedizin Berlin Germany; 5 QUEST Center for Transforming Biomedical Research Berlin Institute of Health (BIH) Berlin Germany; 6 Faculty of Computing, Engineering and the Built Environment School of Computing and Digital Technology Birmingham City University Birmingham United Kingdom; 7 School of Computer Science University College Dublin Dublin Ireland; 8 Information, Communication and Entertainment Research Institute Technological University Dublin Dublin Ireland

**Keywords:** cognitive rehabilitation, Elo rating, predictors, stroke rehabilitation, web-based tasks

## Abstract

**Background:**

Stroke is a worldwide cause of disability; 40% of stroke survivors sustain cognitive impairments, most of them following inpatient rehabilitation at specialized clinical centers. Web-based cognitive rehabilitation tasks are extensively used in clinical settings. The impact of task execution depends on the ratio between the skills of the treated patient and the challenges imposed by the task itself. Thus, treatment personalization requires a trade-off between patients’ skills and task difficulties, which is still an open issue. In this study, we propose Elo ratings to support clinicians in tasks assignations and representing patients’ skills to optimize rehabilitation outcomes.

**Objective:**

This study aims to stratify patients with ischemic stroke at an early stage of rehabilitation into three levels according to their Elo rating; to show the relationships between the Elo rating levels, task difficulty levels, and rehabilitation outcomes; and to determine if the Elo rating obtained at early stages of rehabilitation is a significant predictor of rehabilitation outcomes.

**Methods:**

The PlayerRatings R library was used to obtain the Elo rating for each patient. Working memory was assessed using the DIGITS subtest of the Barcelona test, and the Rey Auditory Verbal Memory Test (RAVLT) was used to assess verbal memory. Three subtests of RAVLT were used: RAVLT learning (RAVLT075), free-recall memory (RAVLT015), and recognition (RAVLT015R). Memory predictors were identified using forward stepwise selection to add covariates to the models, which were evaluated by assessing discrimination using the area under the receiver operating characteristic curve (AUC) for logistic regressions and adjusted R^2^ for linear regressions.

**Results:**

Three Elo levels (low, middle, and high) with the same number of patients (n=96) in each Elo group were obtained using the 50 initial task executions (from a total of 38,177) for N=288 adult patients consecutively admitted for inpatient rehabilitation in a clinical setting. The mid-Elo level showed the highest proportions of patients that improved in all four memory items: 56% (54/96) of them improved in DIGITS, 67% (64/96) in RAVLT075, 58% (56/96) in RAVLT015, and 53% (51/96) in RAVLT015R (*P*<.001). The proportions of patients from the mid-Elo level that performed tasks at difficulty levels 1, 2, and 3 were 32.1% (3997/12,449), 31.% (3997/12,449), and 36.9% (4595/12,449), respectively (*P*<.001), showing the highest match between skills (represented by Elo level) and task difficulties, considering the set of 38,177 task executions. Elo ratings were significant predictors in three of the four models and quasi-significant in the fourth. When predicting RAVLT075 and DIGITS at discharge, we obtained R^2^=0.54 and 0.43, respectively; meanwhile, we obtained AUC=0.73 (95% CI 0.64-0.82) and AUC=0.81 (95% CI 0.72-0.89) in RAVLT075 and DIGITS improvement predictions, respectively.

**Conclusions:**

Elo ratings can support clinicians in early rehabilitation stages in identifying cognitive profiles to be used for assigning task difficulty levels.

## Introduction

### Background

Stroke is currently considered one of the top global causes of disability, with most survivors of stroke in need of inpatient rehabilitation at specialized clinical centers [1]. Recent studies have reported that almost 40% of survivors of stroke sustain cognitive impairment [2]. The World Health Organization definition of cognitive impairment has been recently referred [3] to as *problems experienced by an individual in remembering things, making decisions, learning abilities or concentrating on tasks that affect their everyday life*.

Cognitive rehabilitation (neuropsychological rehabilitation) relies on brain plasticity to induce neuroplastic changes to compensate for cognitive impairments [4]. Brain injury is one of the key causes of cognitive impairment; however, other factors contribute to the ever-increasing number of people in need of cognitive rehabilitation (one of them being the global trend in population aging).

One of the most frequent cognitive problems reported by poststroke patients in their daily lives is related to memory loss [5,6]. To date, associations between factors for ischemic stroke and clinical outcomes have been analyzed predominantly in older rather than younger patients [7]; however, the incidence rates of ischemic stroke have increased in young adults in the United States [8] and also in Europe [9].

New strategies for providing cognitive rehabilitation services are constantly required and are continuously being integrated into clinical practice [10]. One such strategy is the use of web-based systems, and several of these systems have already been used to optimize cognitive interventions [11,12]. However, because of the relatively recent development of these services, the best strategies to integrate them into everyday clinical practice are still unclear [13]. Nevertheless, strategies targeting the personalization of the proposed activities for patients according to their specific needs appear to be more effective [14].

A typical cognitive rehabilitation program mainly provides exercises that require repetitive use of the impaired cognitive system in a progressively more demanding [15] sequence of tasks. The impact of a task or exercise execution depends on the ratio between the skills of the treated patient and the challenges involved in the execution of the task itself. Thus, determining the correct training schedule requires a quite precise trade-off between sufficient stimulation and sufficiently achievable tasks, which is far from trivial and is still an open issue, both empirically and theoretically [16,17].

Furthermore, prediction of specific outcomes after stroke rehabilitation is used by clinicians to improve the accuracy of prognoses, set attainable goals, reach shared decisions, personalize rehabilitation plans, and inform patients and relatives [18].

In this study, we propose the application of Elo ratings to provide clinicians with a ranking of patients at an early stage of cognitive rehabilitation by using the results of web-based cognitive rehabilitation tasks. We hypothesize that (1) such ranking of patients will allow clinicians to match patient’s skills with task difficulties, thereby enabling better treatment personalization, and (2) such a rating will be a significant predictor of patients’ outcomes for memory cognitive function. The original proposal of the Elo rating system was designed to rate chess players, and the rating system was named after its creator Arpad Elo [19].

The Elo system works as follows: an initial rating is assigned to each player every time a player plays a match. This rating is updated for both players depending on the result of the match. If the winner is the player with the higher rating, the update is small, and it is larger depending on how unexpected the victory is, according to their previous ratings [20].

The basic Elo rating system is used in several types of contests beyond chess, for example, football [21]; however, different applications have been extensively reported elsewhere. It has been used for eliciting user preferences in community-based sites [22], assessing security and vulnerability risks [23], ranking posts in web-based forums [24], rating patterns in videogames [25], detecting fabric defects in the textile industry [26], providing students with individualized learning materials in educational settings [20], studying traffic congestion in urban transportation [27], studying dominance hierarchies in behavioral and evolutionary animal ecology [28], forecasting sales and optimizing prices of new product releases [29], allocating resources for criminal justice to support supervision officers [30], and identifying people using facial comparative descriptions [31].

Nevertheless, to the best of our knowledge, Elo ratings have not been applied in cognitive rehabilitation in general or in the specific use–case of a web-based application where patients perform web-based cognitive tasks during their rehabilitation period.

### Objectives

In this study, we propose that instead of considering matches between, for example, chess players, we consider matches between patients and web-based cognitive rehabilitation tasks.

The aims of this study are (1) to demonstrate the feasibility of the approach by presenting a synthetic data set where we obtain an Elo rating for each patient by considering each execution of a cognitive rehabilitation task by the patient as a match between the patient and the task; (2) to obtain the Elo rating of each patient in a real rehabilitation setting where adult patients with ischemic stroke follow cognitive rehabilitation by executing web-based rehabilitation tasks and use these Elo ratings to perform a stratification of patients into 3 groups according to their Elo rating (low, middle, and high); (3) to analyze the relationship among the three Elo rating levels and the proportion of tasks executed at three increasing difficulty levels (1, 2, and 3) with the rehabilitation outcomes in the memory cognitive function; and (4) to develop and internally validate four predictive models for auditory verbal learning memory and working memory outcomes using Elo ratings obtained at early stages of rehabilitation as independent variables and state-of-the-art variables (eg, sex, age, and length of stay). The first two models are developed for predicting auditory verbal learning memory and working memory at discharge and the other two for predicting improvements in auditory verbal learning memory and working memory at discharge.

## Methods

### Participants and Clinical Setting

The setting was the inpatient acquired brain injury rehabilitation unit of the Institut Guttmann hospital, a specialized clinical center certified in quality of care and patient safety (Joint Commission International since 2005 and consecutively recertified in 2009, 2012, and 2018). The initial study population consisted of 344 patients with ischemic stroke who were consecutively admitted for inpatient rehabilitation from March 2009 to September 2019. Patients were included in the study if they had been admitted within 180 days of the onset of an ischemic stroke. Patients who were admitted >180 days after a stroke (31/344, 9%), who had no cognitive assessment within a week after stroke rehabilitation admission (18/344, 5.2%), or had missing data (7/344, 2%) were excluded. Therefore, 83.7% (288/344) of the patients were available for analysis. Patients with aphasia were not included in the n=344 initial sample as they follow a different rehabilitation protocol involving a different set of cognitive assessments and, therefore, need to be analyzed separately (in future work).

At admission, each patient was assigned a physician who coordinated the rehabilitation team (a nurse, a neuropsychologist, a physiotherapist, an occupational therapist, a social worker, and a clinical psychologist based on the characteristics of the case). Therefore, admission and discharge cognitive assessments (as well as all clinical and demographic data analyzed in this study) were systematically recorded in the electronic health records of the hospital. The authors confirm that this study is compliant with the Helsinki Declaration of 1975, as revised in 2008, and it was approved by the Ethics Committee of Clinical Research of Institut Guttmann.

The participants were anonymized and nonidentifiable. A specific written informed consent was not required for participants to be included in this study; nevertheless, at admission to Institut Guttmann, participants provided written informed consent to be included in research studies addressed by the Institut Guttmann hospital.

### Web-Based Cognitive Rehabilitation System

The Guttmann, NeuroPersonalTrainer web-based cognitive rehabilitation platform used in this study comprises a set of 149 different web-based cognitive rehabilitation tasks. There is no established previous order in which patients should execute such tasks. Therefore, every patient executed (eventually) a different subset of them in a different order during their rehabilitation process, taking between 2 and 6 months, distributed over two to five sessions a week. During each session, the patient executed between 4 to 10 cognitive rehabilitation tasks, and the total duration of one session ranged between 45 minutes to 1 hour. Each task mainly addressed one of the following functions: memory, executive functioning, attention, gnosias, calculus, orientation, language, and social cognition. Immediately after each execution of a task, the patient received a feedback on performance (ranging from 0-100, as the percentage of compliance), with 0% being the lowest level of compliance and 100% being the highest.

### Cognitive Assessments at Admission

Before starting web-based cognitive rehabilitation using the Guttmann, NeuroPersonalTrainer platform, every patient was assessed once using standardized tests specifically validated for the population under study. Specific linguistic abilities were assessed using three subtests of the Barcelona test [32,33]: (1) repetition (maximum score=10), (2) denomination (maximum score=14), and (3) comprehension (maximum score=16). For assessing verbal fluency, the phonetic verbal fluency test [34] was used. The Trail Making Test was used to assess executive functioning [35] and the Wechsler Adult Intelligence Test–III [36] to assess visuospatial construction and perception.

### Cognitive Assessments at Admission and Discharge: Memory Variable

In this study (without loss of generality), we assessed improvements in the memory cognitive function using the Rey Auditory Verbal Memory Test (RAVLT) [37] and the DIGITS subtest of Barcelona test [32]. RAVLT comprises three subtests: RAVLT learning (RAVLT075), free-recall memory (RAVLT015), and recognition (RAVLT015R). In RAVLT075, the patient was asked to recall as many words as possible from a list of 15 words, repeated five times. After a latency of 20 minutes, the patient was asked to recall the words (RAVLT015), and then the patient heard a list of 50 words containing the 15 initial sets that had to be recognized by the patient (RAVLT015R).

The DIGITS subtest (direct version) of the Barcelona test addresses working memory, and the patient was asked to repeat a series of numbers of variable lengths (3-9) until they failed in two consecutive series, reporting the largest series before failure [32].

### Elo Rating Formulation

The Elo rating system [19] is formally defined as [20]: given a rating estimate *θ_i_* for each player *i*, the result of a match between players *i* and *j* is represented by R*_ij_*∈{0,1}.

The actual ratings of each player are used to estimate the probability that player *i* wins:



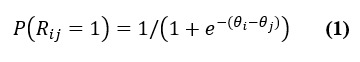



which is used to update the ratings as follows, based on the Bradley-Terry model [38]:



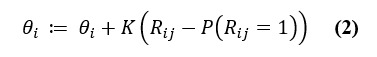



where K is a constant parameter that controls how quickly *θ_i_* changes, with large K values resulting in *θ_i_* changing quickly and small K values resulting in *θ_i_* changing slowly. In this study, we considered three extensions to the original Elo system: Glicko [39], Glicko-2 [40], and Stephenson [41]. Glicko models introduce a measure of reliability to assess the accuracy of the rating; that is, the rating deviation. Stephenson rating can be of interest in our context as it introduces a parameter that considers the strengths of the opponents, [41] being in our case, player *i,* the patient, and player *j,* the cognitive rehabilitation task.

### Regression Models

#### Overview

Demographic and clinical state-of-the-art variables such as age, gender, marital status, and variables related to the rehabilitation program, such as the time in between the onset of stroke and initiation of the rehabilitation program or length of stay, were considered as candidate predictors. Categorical variables were dichotomized: female=0, male=1; low level of education=0, high level of education=1 (depending on the number of years of education); married=1, not married=0. Forward stepwise selection was used to add covariates to the models, which were evaluated by assessing discrimination using area under the receiver operating characteristic curve (AUC), accuracy, sensitivity, and specificity for logistic regressions and to maximize R^2^ and adjusted R^2^ for linear regressions. The variance inflation factor and tolerance (1/variance inflation factor) were used to test the multicollinearity of independent variables (tolerance ≤0.40 indicates a multicollinearity problem) [42]. The Durbin-Watson (D-W) test was used to assess the assumption of independent errors (D-W should be close to 2 to meet the assumption of independence [42]). The Elo rating algorithm calculations (including Glicko, Glicko-2, and Stephenson) were applied using the PlayerRatings R package [41]. R v3.5.1 (R Foundation for Statistical Computing) was used for all statistical analyses. The level of significance was set at *P*=.05.

#### Dependent Variables

In linear regressions, the dependent variables were RAVLT075 and DIGITS at discharge. In logistic regressions, the aim was to predict improvement in RAVLT075 (if RAVLT075 at discharge–RAVLT075 at admission ≥5, then improvement=true; else, improvement=false) and improvement in DIGITS (if DIGITS at discharge–DIGITS at admission ≥1, then improvement=true; else, improvement=false).

## Results

### Demographic Characteristics and Cognitive Assessments

Table 1 shows the demographic characteristics and clinical assessments of the 288 included patients.

The mean age at the time of the lesion was 51 (SD 9) years. The proportion of participants aged <65 years was 93.8% (270/288) (as opposed to most studies addressing ischemic stroke, we analyzed working-age participants). In relation to sex, in our data set, the proportion was 67.7% (195/288) men and 32.3% (93/288) women, which seems to suggest a bias in favor of men. Nevertheless, it somehow reflects reality in the general population, where the proportion of men experiencing ischemic stroke is larger than that of women [43-45]; however, women experience more hemorrhagic strokes [46].

**Table 1:** Demographics and clinical assessments (N=288)^a^.

Variables			Admission		Discharge
Age (years), mean (SD)			51 (9)		N/A^b^
Age <65 years, n (%)			270 (93.8)		N/A
Males, n (%)			195 (67.7)		N/A
Marital status (married), n (%)			180 (62.5)		N/A
**Educational level, n (%)**					
	Read and write	9 (3.1)		N/A	
	Primary	114 (50)		N/A	
	Secondary	88 (30.5)		N/A	
	Higher	66 (22.9)		N/A	
NIHSS^c^, median (IQR)			11 (7-15)		N/A
TMT^d^ A, mean (SD)			82 (64)		N/A
TMT B, mean (SD)			157 (90)		N/A
PMR^e^, mean (SD)			27 (12)		N/A
VC^f^–CUBS, mean (SD)			23 (12)		N/A
VP^g^-IMAGES, mean (SD)			17 (3)		N/A
VP–WAIS III^h^, mean (SD)			37 (15)		N/A
Barcelona test–repetition, mean (SD)			9 (1)		N/A
Barcelona test–denomination, mean (SD)			13 (1)		N/A
Barcelona test–comprehension, mean (SD)			15 (1)		N/A
Barcelona test–DIGITS, mean (SD)			3 (1)		4 (1)
Barcelona test–DIGITS, median (IQR)			4 (3-4)		4 (3-5)
RAVLT^i^ 075, mean (SD)			37 (10)		43 (11)
RAVLT075, median (IQR)			37 (30-45)		44 (35-52)
RAVLT015, mean (SD)			6 (3)		8 (3)
RAVLT015, median (IQR)			7 (5-9)		9 (6-12)
RAVLT015R, mean (SD)			10 (4)		11 (3)
RAVLT015R, median (IQR)			12 (8-14)		13 (10-14)
Length of stay (days), mean (SD)			88 (36)		N/A
Length of stay (days), median (IQR)			84 (55-113)		N/A
Time since onset to rehab admission (days), mean (SD)			55 (35)		N/A
Time since onset to rehab admission (days), median (IQR)			43 (29-75)		N/A

^a^Results are presented as mean (SD), median (IQR), or percentage, when appropriate.

^b^N/A: not applicable.

^c^NIHSS: The National Institutes of Health Stroke Scale.

^d^TMT: Trail Making Test.

^e^PMR test assesses the capacity of word generation according to an initial letter (P, M, and R).

^f^VC: visual construction.

^g^VP: visual perception.

^h^WAIS-III: Wechsler Adult Intelligence Test–III.

^i^RAVLT: Rey Auditory Verbal Memory Test.

### Elo Rating: Feasibility Case

**Figure 1:**
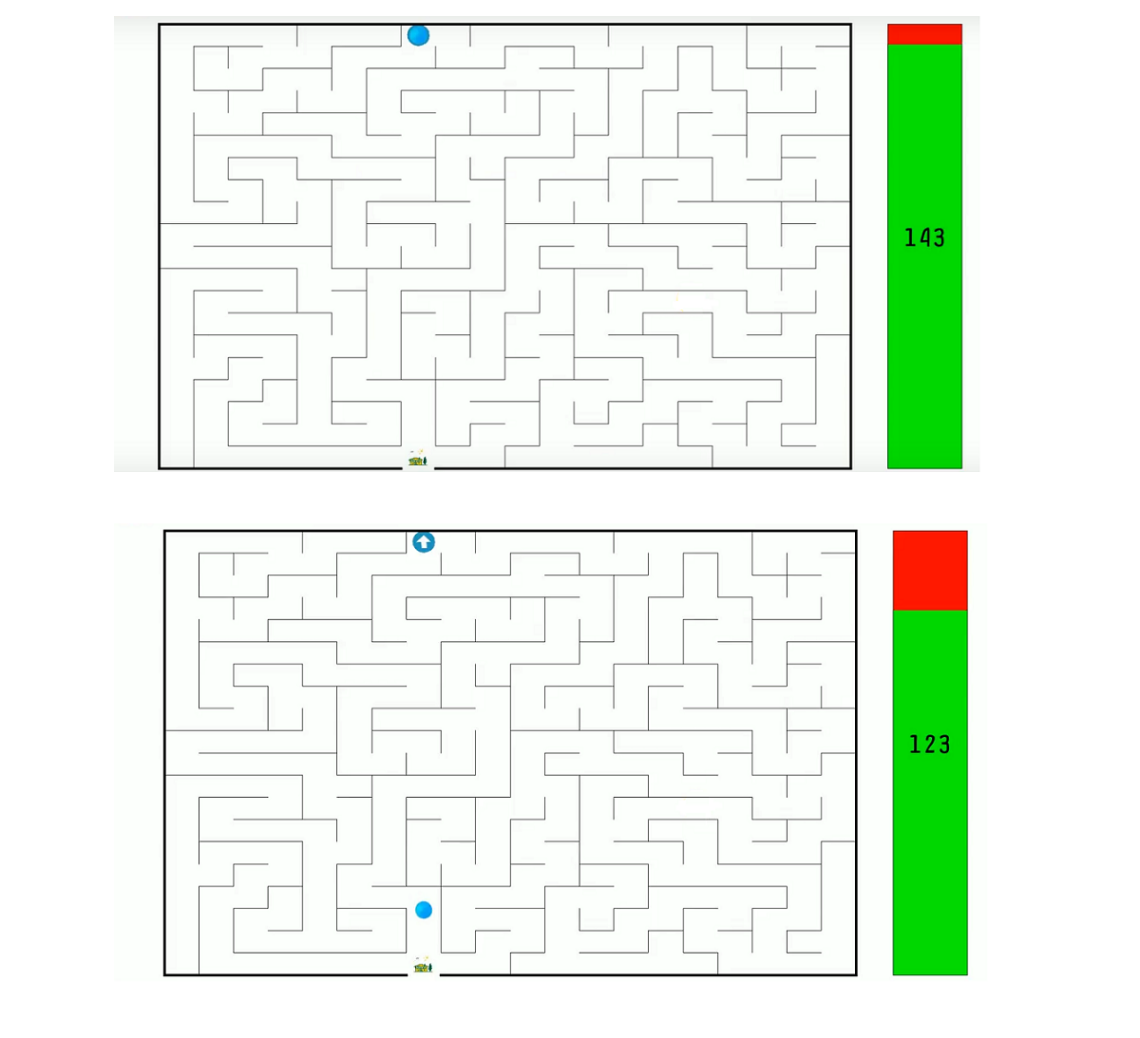
Two screenshots of the maze task, showing the initial position of the blue ball (top) and its position at the end of the task (bottom).

We initially ran the four different Elo rating approaches (standard Elo, Glicko, Glicko-2, and Stephenson) in a reduced data set of 20 patients, each of whom executed the same task 20 times. Two screenshots of the selected task are presented in Figure 1. The task addresses executive functioning (planning), and the objective is to move a blue ball from an initial position in a maze to the final position, minimizing the number of moves. The bar at the right indicates the time left to perform the task. Figure 1 top shows the initial position of the ball, and Figure 1 bottom shows the status 20 seconds later when the objective was accomplished.

The included 20 patients were stratified into three categories according to their compliance in the maze task as follows:

- low compliance={id1, id2, id3, id4, id5, id6};
- mid compliance={id7, id8, id9, id10, id11, id12, id13};
- high compliance={id14, id15, id16, id17, id18, id19, id20}.

**Figure 2:**
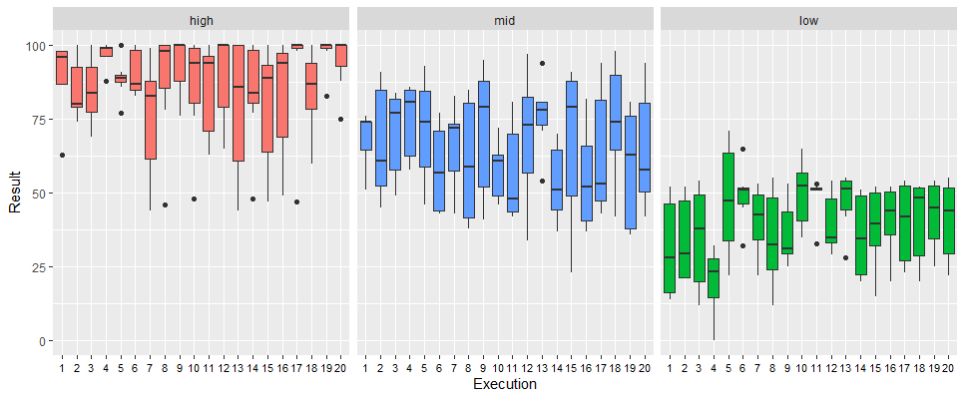
Boxplots of the obtained results in the maze task at each execution in the 3 groups, showing the high, middle, and low levels of compliance in the task.

Figure 2 presents the boxplots of the obtained results in the maze task at each execution in the 3 groups, showing their different levels of compliance.

We then ran the four Elo rating systems with default values for the initial ratings and K. We considered that when a patient gets a result >50%, they win the match against the maze; however, if their result is <50%, the maze wins. The ratings obtained using the Glicko approach are presented in Table 2. Patients are ordered in Table 2 according to their obtained ratings. Table 2 shows patients id19, id20, id16, id14, id17, id15, and id18 at the first seven positions. Similarly, patients from the midcompliance group are in positions 8-14, and patients from the low compliance group are in the bottom positions. The maze task itself is also considered as a player; it played all 400 matches, winning 118 and losing 282.

**Table 2:** Glicko ratings after 20 executions of the maze task (n=20 synthetic patients).

Player	Glicko rating (deviation)	Games	Win	Loss
id19	2565 (146.26)	20	20	0
id20	2565 (146.26)	20	20	0
id16	2450 (124.22)	20	19	1
id14	2368 (118.61)	20	18	2
id17	2364 (124.24)	20	18	2
id15	2293 (113.49)	20	17	3
id18	2273 (119.90)	20	17	3
id12	2240 (104.33)	20	16	4
id10	2195 (97.57)	20	15	5
id13	2190 (99.44)	20	15	5
id9	2177 (103.78)	20	15	5
id11	2143 (95.22)	20	14	6
id7	2122 (101.43)	20	14	6
id8	2112 (91.53)	20	13	7
id3	2035 (86.81)	20	10	10
Maze	1999 (36.77)	400	118	282
id2	1981 (87.77)	20	9	11
id5	1965 (88.01)	20	9	11
id4	1960 (87.11)	20	8	12
id6	1957 (87.46)	20	8	12
id1	1930 (89.33)	20	7	13

**Figure 3:**
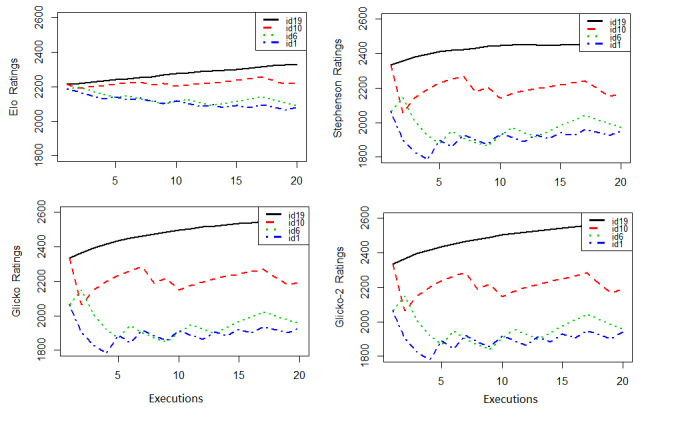
Elo ratings using all four approaches (traditional Elo, Stephenson, Glicko, and Glicko-2) for patient representatives of each of the compliance groups; id1 and id6 (low level), id10 (midlevel) and id19 (high level).

Figure 3 shows the obtained ratings using all four approaches for patient representatives of each of the compliance groups; we plotted id1 and id6 patients from the low-level group, id10 from the midlevel group, and id19 from the high level of compliance group to visualize how the Elo ratings represented their compliance levels.

### Cognitive Task Executions in Guttmann, NeuroPersonalTrainer Platform

#### Overview

Table 3 summarizes all task executions during the whole rehabilitation process for all 288 included patients. A total of 44,814 task executions were performed in 5088 sessions during the period under study. Each patient performed 155 task executions on average. When considering the different functions addressed by the tasks, the most frequently executed were those addressing memory (18,183 executions), comprising almost 40.57% (18,183/44,814) of the total executions.

**Table 3:** Cognitive rehab task executions (N=288 patients).

Description	Values
Total number of task executions	44,814
Executions per patient, mean (SD)	155 (113.2)
Total number of sessions	5008
Sessions executed per patient, mean (SD)	17 (11.5)
Tasks executed per session per patient, mean (SD)	9 (4.4)
Total number of memory tasks executed	18,183
Total number of executive functioning tasks executed	14,061
Total number of attention tasks executed	8062
Total number of gnosias tasks executed	1795
Total number of calculus tasks executed	1695
Total number of orientation tasks executed	741
Total number of language tasks executed	261
Total number of social cognition tasks executed	16
Memory task results, mean (SD)	53.1 (36.4)
Executive functioning tasks results, mean (SD)	49.6 (38.7)
Attention task results, mean (SD)	59.4 (36.7)
Gnosias task results, mean (SD)	74.4 (30.8)
Calculus task results, mean (SD)	72.9 (35.8)
Orientation task results, mean (SD)	75.6 (38.0)
Language task results, mean (SD)	55.5 (38.4)
Social cognition task results, mean (SD)	56.7 (37.1)

#### Preprocessing: Removing Less Executed Tasks

As introduced in the section *Web-Based Cognitive Rehabilitation System*, the Guttmann, NeuroPersonalTrainer cognitive platform includes 149 different web-based tasks. There is no established previous order or frequency in which patients should execute such tasks; therefore, in this section, we analyze task execution frequencies. As shown in Table S1 (Multimedia Appendix 1), several tasks were very infrequently executed. As detailed in Table S2 (Multimedia Appendix 1), 68 tasks accounted for 38,177 executions. Therefore, 45.6% (68/149) of all available tasks accounted for 85.18% (38,177/44,814) of all executions. In this section, we analyzed these 68 tasks (executed by all N=288 patients) and stratified them into three difficulty levels, considering their input parameter configurations during the 38,177 executions.

### Ranking Patients Using the Initial 50 Task Executions: Elo Rating

We used the Stephenson rating with default parameters, considering the following criteria:

- If the result ≤39%, then the task wins.
- If 40% ≤ result ≤ 64%, then the result is a draw.
- If the result ≥65%, then the patient wins.

The Stephenson ratings were obtained by considering the first 50 task executions for every patient. We then stratified all 288 patients into 3 groups (each group comprised n=96 patients), according to their Elo ratings (low, middle, and high). Table 4 shows the memory assessments at admission and discharge, percentage of patients that improved, mean number of executed tasks, and obtained result comparisons for the three Elo levels (low, mid, and high) obtained using the 50 initial task executions for n=288 patients, with 96 patients in each Elo group that performed 38,177 task executions of the most frequent 68 tasks during rehabilitation.

**Table 4:** Memory assessments at admission and discharge, percentage of patients that improved, mean number of executed tasks, and obtained results comparisons for the three Elo levels (low, middle, and high) obtained using the 50 initial task executions (N=288 patients, 96 patients in each Elo group that executed 38,177 tasks during rehabilitation).

Variables		Low Elo (n=12,431)	Mid Elo (n=12,449)	High Elo (n=13,297)	*P* value
Sex (female), n (%)		4396 (35.36)	4607 (37.01)	3793 (28.52)	<.001
Age (years) when starting rehabilitation, mean (SD)		52 (8)	51 (8)	48 (10)	<.001
DIGITS at admission, mean (SD)		3.4 (0.9)	3.8 (0.9)	4.0 (1.0)	<.001
RAVLT^a^ 075 at admission, mean (SD)		37 (9)	36 (10)	38 (10)	<.001
RAVLT015 at admission, mean (SD)		6 (3)	6 (3)	7 (3)	<.001
RAVLT015R at admission, mean (SD)		10 (4)	10 (4)	11 (4)	<.001
Length of stay (days), mean (SD)		104 (37)	105 (35)	106(40)	.05
Executed tasks, mean (SD)		245 (129)	222 (122)	241 (124)	<.001
Obtained results in tasks, mean (SD)		37 (36)	56 (36)	68 (33)	<.001
DIGITS at discharge, mean (SD)		3.5 (1.0)	4.5 (0.9)	4.4 (0.8)	<.001
RAVLT075 at discharge, mean (SD)		42 (11)	45 (11)	45 (12)	<.001
RAVLT015 at discharge, mean (SD)		8 (3)	9 (3)	9 (4)	<.001
RAVLT015R at discharge, mean (SD)		11 (3)	12 (3)	12 (3)	<.001
DIGITS IMP^b^ (yes), n (%)		2859 (22.99)	7059 (56.7)	5353 (40.26)	<.001
RAVLT075 IMP (yes), n (%)		5308 (42.69)	8356 (67.12)	7484 (56.28)	<.001
RAVLT015 IMP (yes), n (%)		6136 (49.36)	7325 (58.84)	7482 (56.26)	<.001
RAVLT015R IMP (yes), n (%)		6802 (54.72)	6683 (53.68)	7132 (53.64)	<.001
**Task difficulty level, n (%)**					
	Level 1	4812 (38.71)	3997 (32.11)	3536 (26.59)	<.001
	Level 2	3999 (32.17)	3857 (30.98)	4346 (32.68)	<.001
	Level 3	3620 (29.12)	4595 (36.91)	5415 (40.72)	<.001

^a^RAVLT: Rey Auditory Verbal Learning Test.

^b^IMP: improved.

### Importance of Elo Rating in Predicting Outcomes: RAVLT075 and DIGITS

Table 5 presents the obtained predictors of RAVLT075 at discharge (model 1), 54% of the variance explained and the obtained predictors of DIGITS at discharge (model 2), 43% of the variance explained.

When the Elo rating feature is excluded from model 1, it explains 52% of the variance, and when it is excluded from model 2, the resulting model explains 42%.

**Table 5:** Multivariate linear regressions, nonstandard β (95% CI), standard β, Durbin-Watson (D-W) test, variance inflation factor, and R^2^ and adjusted R^2^ for RAVLT075 and DIGITS at discharge (N=288).

Variables		β (95% CI)	Standard β	1/VIF^a^	*P* value	R^2^	Adjusted R^2^
**Model 1 predictors of RAVLT^b^ 075 at discharge**							
	Elo rating	.01 (.01 to .02)	.09	0.95	.02	0.55	0.54
	RAVLT075 at admission	.76 (.67 to .85)	.66	0.92	<.001	0.55	0.54
	LOS^c^	.04 (.01 to .06)	.13	0.98	.002	0.55	0.54
	Sex	−2.48 (−4.48 to −0.48)	–0.10	0.93	.01	0.55	0.54
	Age (years)	−0.09 (−0.19 to .01)	–0.07	0.92	.06	0.55	0.54
	D-W^d^=1.89	N/A^e^	N/A	N/A	.37	0.55	0.54
**Model 2 predictors of DIGITS at discharge**							
	Elo rating	.00 (.00 to .00)	.10	0.88	.02	0.44	0.43
	DIGITS at admission	.63 (.54 to .72)	.63	0.91	<.001	0.44	0.44
	LOS	.00 (.00 to .00)	.05	0.98	.22	0.44	0.44
	Sex	.04 (−0.15 to .23)	.01	0.95	.67	0.44	0.44
	Age (years)	.00 (.00 to .01)	.02	0.94	.53	0.44	0.44
	D-W=2.01	N/A	N/A	N/A	.95	0.44	0.44

^a^VIF: variance inflation factor.

^b^RAVLT: Rey Auditory Verbal Memory Test.

^c^LOS: length of stay.

^d^D-W: Durbin-Watson test.

^e^N/A: not applicable.

### Importance of Elo Rating in Predicting Improvement: RAVLT075 and DIGITS

Table 6 presents the models used for predicting improvement in RAVLT075 and DIGITS. We used the criteria to decide whether a patient improved as described in the *Dependent Variables* section; 50.6% (146/288) of patients improved in RAVLT075, and 34% (98/288) of patients improved in DIGITS. We used the same Elo ratings as described in the *Ranking Patients Using the Initial 50 Task Executions: Elo Rating* section. Model 3 yielded an AUC of 0.73 (95% CI 0.64-0.82) for improvement in RAVLT075, with an accuracy=0.64 (95% CI 0.54-0.72), specificity=0.55, and sensitivity=0.73. Model 4 yielded an AUC of 0.81 (95% CI 0.72-0.89) for improvement in DIGITS, with an accuracy=0.73 (95% CI 0.64-0.81), specificity=0.22 and sensitivity=0.97. Models 3 and 4 are detailed in Table 6. When the Elo rating was excluded as an independent variable for model 3, the model yielded an AUC of 0.66 (95% CI 0.56-0.76) for improvement in RAVLT075, with an accuracy=0.62 (95% CI 0.52-0.71), specificity=0.62, and sensitivity=0.62. When the Elo rating was excluded as an independent variable for model 4, the model yielded an AUC of 0.73 (95% CI 0.62-0.83) for improvement in DIGITS, with an accuracy=0.72 (95% CI 0.62-0.80), specificity=0.34, and sensitivity=0.92. As shown in Table S3 (Multimedia Appendix 1), RAVLT075 was highly correlated with RAVLT015 and RAVLT015R at admission and at discharge.

**Table 6:** Multivariable logistic regressions, nonstandard β, odds ratio (95% CI), variance inflation factor for RAVLT075, and DIGITS improvement at discharge (N=288).

Variables			Odds ratio (95% CI)		β coefficients		1/VIF^a^		*P* value
**Model 3^b^ predictors of RAVLT^c^ 075 improvement at discharge**									
	Rating	1.00 (1.00-1.00)		.61		0.93		.02	
	RAVLT075 at admission	0.95 (0.92-0.97)		−0.95		0.88		<.001	
	LOS^d^	1.00 (1.00-1.01)		.67		0.98		<.001	
	Sex	0.64 (0.37-1.11)		−0.40		0.92		.12	
	Age	0.97 (0.94-0.99)		−0.52		0.92		.04	
**Model 4^e^ predictors of DIGITS improvement at discharge**									
	Rating	1.00 (0.99-1.00)		.57		0.86		.06	
	DIGITS at admission	0.38 (0.26-0.52)		−2.04		0.86		<.001	
	LOS	1.00 (1.00-1.01)		.71		0.97		.01	
	Sex	1.02 (0.57-1.85)		.02		0.96		.92	
	Age	1.00 (0.97-1.02)		.01		0.95		.95	

^a^VIF: variance inflation factor.

^b^Area under the receiver operating characteristic curve=0.73 (95% CI 0.64-0.82), accuracy=0.64 (95% CI 0.5451-0.7281), specificity=0.55, and sensitivity=0.73.

^c^RAVLT: Rey Auditory Verbal Memory Test.

^d^LOS: length of stay.

^e^Area under the receiver operating characteristic curve=0.81 (95% CI 0.72-0.89), accuracy=0.73 (95% CI 0.64-0.81), specificity=0.22, and sensitivity=0.97.

## Discussion

### Principal Findings

To the best of our knowledge, in this study, Elo ratings were applied in the context of web-based cognitive rehabilitation tasks for the first time. We demonstrated the feasibility of using Elo ratings by using a publicly available R library (PlayerRatings) [41] on a synthetic use–case of 20 patients executing one task 20 times.

We then obtained the Elo ratings for each patient in a real rehabilitation setting where 288 adult patients with ischemic stroke followed cognitive rehabilitation, executing 68 different web-based rehabilitation tasks 38,177 times. We then performed a stratification of the patients into 3 groups (96 patients each) according to their Elo rating (low, middle, and high). We have shown the relationships among the three Elo rating levels and the proportion of tasks executed at three increasing difficulty levels (1, 2, and 3) with the rehabilitation outcomes in the memory cognitive function. We then developed four predictive models, where the Elo rating variables were significant in three of them (and quasi-significant in the fourth) for auditory verbal learning memory and working memory outcomes. We found that including Elo ratings as independent variables increased the model performance (for both linear and logistic regressions).

### Clinical Implications

Several web-based cognitive rehabilitation platforms integrate some kind of stratification of patients as an initial step for treatment personalization. The web-based platform used in this study integrates an automatic therapy planning functionality—the intelligent therapy assistant (ITA) [47]. The ITA takes a set of patients’ cognitive profiles as the starting point, obtained using cluster analysis on the baseline cognitive evaluation. When a new patient starts cognitive training in Guttmann, NeuroPersonalTrainer, the ITA dynamically assigns the patient to the appropriate cluster. The ITA then schedules different cognitive tasks during a user-defined rehabilitation period for the new patient. Therefore, an important clinical implication of our results in this study involves the ITA (or any other data-driven therapy assistant) starting point: using Elo rating as a starting point, alternative to cluster analysis.

Obtaining an initial Elo rating for each patient is a simple process (in terms of both implementation and interpretation of results). As remarked in previous research, for example, in the field of educational tutoring systems, Elo rating use is encouraged because of its simplicity [20]. As shown in Table 3, the mean number of tasks executed by a patient in a session is 9, so in about five sessions (usually 2 weeks), an Elo rating for each patient obtained using the first 50 task executions will be available.

Therapists can then use the Elo rating to assign the patient to a skill level. In this study, in Table 4, we present the results using three skill levels, each of them with the same number of patients (96; or one-third of the N=288 total participants). Table 4 shows that, for example, 67% (64/96) of patients in the mid-Elo group improved in the RAVLT075 item, and 58% (56/96) of patients in the mid-Elo group improved in the RAVLT015 item. Meanwhile, for example, only 23% (22/96) of patients in the low Elo group improved in the DIGITS item. The low Elo group performed 29.1% (3,617/12,431) of their tasks at difficulty level 3, whereas the mid-Elo group performed 31% (3,859/12,449) of their tasks at difficulty level 2. This seems to suggest that patients in the low Elo group could have performed a higher proportion of tasks at difficulty level 1, which is more appropriate to their skills. Patients in the mid-Elo group performed a higher proportion of tasks according to their skill levels, which seems to be related to a higher proportion of patients obtaining improvements in the four memory items presented in Table 4.

Another clinical implication was noted on in a recent systematic review on computerized cognitive training [48]. The review highlighted the need to develop interventions focused on specific cognitive functions by means of concrete training or rehabilitation activities (or tasks). Our results contribute in that sense; considering, for example, model 1 for predicting RAVLT075 at discharge, we obtained a standard β=.09 for the Elo rating variable. Therefore, for every 113 points obtained in the Elo rating, an extra point in RAVLT075 at discharge is obtained. If we consider, for example, in the maze task presented in Figure 1, patient id12 (Elo ranking=2240) and patient id8 (Elo ranking=2112) presented in Table 2, the difference between their Elo ratings is 128 points, with both patients belonging to the intermediate compliance group. Similar Elo rating scores were obtained for the final sample of N=288. Therefore, therapists can identify at the early stages of the rehab process–specific cognitive tasks where patients are close to obtaining a draw or a win (result ≥40%) and address different strategies [48] to improve performance in such specific tasks.

### Limitations of This Study

Several limitations to the study need to be highlighted. First, we conducted a single-center study, an advantage of which is that data were obtained and included by clinicians trained in neurological rehabilitation, and all patients were managed under the same stroke rehabilitation protocols. The Guttmann, NeuroPersonalTrainer platform has already been integrated into the clinical practice of several acquired brain injury centers; nevertheless, their patients were not included in this analysis. A multicenter stroke study may include an initial preprocessing phase, wherein patients are grouped according to their initial National Institutes of Health Stroke Scale severity to avoid additional heterogeneity. Thereafter, Elo rating techniques, such as those proposed in this study, maybe applied within such groups. External validation assessments common to all participating centers are also an important aspect to be addressed in this future multicenter study. Second, the studied health area belongs mainly to the urban population, with a small rural population or populations from other regions. Third, our analysis lacked computerized tomography or magnetic resonance imaging examinations that describe the presence of contusion, hematoma, hemorrhage, ischemia, or other signs of parenchymal lesions in the frontal, temporal, parietal, occipital, and cerebellar lobes or diffuse axonal injury.

Fourth, our sample did not include any patients with missing data. All data used as inputs were complete. Fifth, our analysis did not include indicators of mental health or other comorbidities. Persons who experience a stroke may have one or more preexisting medical comorbidities at the time of injury (eg, alcohol use and depression). Therefore, we plan to include comorbidity analyses in future research studies. Sixth, in all our Elo rating calculations, we used the default value for the K constant. Several approaches to K optimization have been reported, such as hill climbing, gradient descendent, or Bayesian [20], which can also be addressed in future work. Finally, the criteria for defining wins, draws, and losses in our Elo ratings were also constant for every task, and another possible improvement could be to fit such criteria according to the task difficulty level, considering the strength of the opponents (patients’ skills and task difficulty levels) that can be addressed using the Stephenson extension [41].

### Comparison with Prior Work

Cluster analysis has been extensively proposed in previous research to address heterogeneity in patients with acquired brain injury [49-51] and as an initial step for patient profiling. Most previous studies use commercial software products for cluster analysis, which are, in turn, not integrated into the web-based cognitive rehabilitation platform.

In a recent study, Faria et al [52] presented a framework for the creation of personalized cognitive rehabilitation tasks based on a participatory design strategy. They selected 11 paper-and-pencil tasks from standard clinical practice and parameterized them with multiple parameter configurations. A modeling approach was used to quantitatively determine how the task parameters affect each of the cognitive domains (memory, executive functions, attention, and language). For modeling this relationship, the parameters of each task were used as predictors of the demands in each cognitive domain. In our case, the parameters of each task were used by experts to assign a difficulty level to each task (difficulty level 1, 2, and 3, as presented in Table 4), where each task aims to address one main cognitive domain (memory, executive functions, attention, and language).

### Conclusions

We have shown the feasibility of Elo ratings for identifying patients’ profiles at the early stages of cognitive rehabilitation in a real clinical setting. Elo ratings can be used to match skills with task difficulties, aiming to maximize improvements in specific cognitive functions. Such Elo ratings are also significant in predicting cognitive outcomes. Elo ratings increased the models’ performance (for both linear and logistic regressions). Generalization of the use of Elo ratings beyond patients with stroke to any other population with acquired brain injury requiring cognitive rehabilitation in any web-based platform is straightforward because of the simplicity of existing open-access Elo rating implementations.

## Appendix

Multimedia Appendix 1 All initial 44,814 web-based task executions, selection of the most frequently executed 68 tasks (38,177 executions), and correlation analysis of the Rey Auditory Verbal Memory Test and DIGITS assessments.

## Abbreviations

AUC: area under the receiver operating characteristic curve
ITA: intelligent therapy assistant
RAVLT: Rey Auditory Verbal Memory Test
