# Supplementary material for: Toward Personalized Web-Based Cognitive Rehabilitation for Patients With Ischemic Stroke: Elo Rating Approach
Source: JMIR Med Inform. 2021 Nov 10;9(11):e28090. doi: 10.2196/28090 (PMC8663500; doi:10.2196/28090)
Supplement: Multimedia Appendix 1 [file medinform_v9i11e28090_app1.docx]

**Supplementary material.**

Table S1. Total number of executions of all tasks

| **Task identifier** | **number of executions** |
| --- | --- |
| braindamage.lenguaje.comprensionauditiva.semantica.asociarporcategoria | 1 |
| braindamage.socialcognition.cognitivebiases.carasprogresivas | 1 |
| braindamage.socialcognition.cognitivebiases.historiasimposibles | 1 |
| braindamage.socialcognition.cognitivebiases.vinetasbade | 1 |
| braindamage.socialcognition.teoriamente.consejoenlacafeteria | 1 |
| braindamage.socialcognition.teoriamente.laamigametepatas | 1 |
| braindamage.socialcognition.teoriamente.videosironiaysarcasmo | 1 |
| braindamage.lenguaje.escritura.palabras.copiadiferida | 2 |
| braindamage.socialcognition.emotionalprocessing.descubreemocion | 2 |
| braindamage.socialcognition.emotionalprocessing.emocionenvoz | 2 |
| braindamage.socialcognition.emotionalprocessing.pidemasinformacion2 | 2 |
| braindamage.socialcognition.teoriamente.elamigosarcastico | 2 |
| braindamage.socialcognition.teoriamente.vinetascomic | 2 |
| braindamage.lenguaje.comprensionauditiva.palabras.paloidaimagen | 3 |
| braindamage.lenguaje.comprensionauditiva.palabras.paloidaimagenpalescrita | 3 |
| braindamage.lenguaje.comprensionauditiva.semantica.clasificacionimagenes | 3 |
| braindamage.lenguaje.comprensionauditiva.habilidadesbasicas.imagenesidenticas | 5 |
| braindamage.lenguaje.comprensionauditiva.habilidadesbasicas.sonidosentorno | 5 |
| braindamage.lenguaje.lectura.palabras.palabrasidenticas | 5 |
| braindamage.lenguaje.escritura.palabras.unirfragmentos | 6 |
| braindamage.lenguaje.lectura.palabras.ordserautomaticas | 6 |
| braindamage.lenguaje.lectura.palabras.palabraescritaoida | 6 |
| braindamage.lenguaje.comprensionauditiva.semantica.asociarporrelacion | 7 |
| braindamage.lenguaje.lectura.palabras.identificargrafema | 7 |
| braindamage.lenguaje.escritura.palabras.copiadirecta | 8 |
| braindamage.lenguaje.lectura.palabras.clasificar | 8 |
| braindamage.lenguaje.lectura.palabras.eliminardiferente | 8 |
| braindamage.lenguaje.lectura.palabras.palabrasmayusminus | 8 |
| braindamage.lenguaje.comprensionauditiva.habilidadesbasicas.memoriaordentemporal | 9 |
| braindamage.lenguaje.comprensionauditiva.habilidadesbasicas.copiarserie | 10 |
| braindamage.lenguaje.comprensionauditiva.habilidadesbasicas.continuarserie | 11 |
| braindamage.lenguaje.comprensionauditiva.habilidadesbasicas.ordenarportamano | 13 |
| braindamage.lenguaje.comprensionauditiva.palabras.series2palabras | 13 |
| braindamage.lenguaje.comprensionauditiva.semantica.eliminardiferente | 13 |
| braindamage.lenguaje.lectura.palabras.antonimos | 13 |
| braindamage.lenguaje.lectura.palabras.palabrasrelacionadas | 18 |
| braindamage.lenguaje.escritura.palabras.denominacionrespondiendo | 24 |
| braindamage.lenguaje.escritura.palabras.denominacionimagenes | 46 |
| braindamage.memoria.verbal.frases.secuencial.recuerdo.escriure | 46 |
| braindamage.memoria.verbal.text.record.recordescriure | 48 |
| braindamage.memoria.verbal.assocparpalb.pressimul.record.ordrealeat | 59 |
| braindamage.memoria.verbal.assocparpalb.presseque.record.ordrealeat | 61 |
| braindamage.memoria.verbal.assocparpalb.pressimul.record.mateixordre | 61 |
| braindamage.memoria.verbal.assocparpalb.presseque.record.mateixordre | 62 |
| braindamage.memoria.verbal.apreserpalb.presseque.record.difcatg | 73 |
| braindamage.memoria.verbal.frases.simultaneo.recuerdo.escriure | 77 |
| braindamage.memoria.verbal.apreserpalb.pressimul.record.difcatg | 92 |
| braindamage.memoria.visual.associmgpalb.secuencial.recuerdo.ordrealeat | 96 |
| braindamage.memoria.verbal.apreserpalb.pressimul.record.mateixcatg | 104 |
| braindamage.memoria.visual.associmgpalb.secuencial.recuerdo.mateixordre | 104 |
| braindamage.memoria.visual.associmgpalb.simultanea.recuerdo.ordrealeat | 106 |
| braindamage.memoria.verbal.assocparpalb.pressimul.reconeixm.decideix | 108 |
| braindamage.memoria.verbal.apreserpalb.presseque.record.mateixcatg | 111 |
| braindamage.memoria.verbal.apreserpalb.pressimul.reconeixm.decideix | 111 |
| braindamage.memoria.verbal.text.record.recordvf | 119 |
| braindamage.memoria.verbal.frases.secuencial.recuerdo.verdaderofalso | 121 |
| braindamage.memoria.verbal.assocparpalb.presseque.reconeixm.decideix | 127 |
| braindamage.memoria.verbal.assocparpalb.presseque.reconeixm.selecciona | 127 |
| braindamage.memoria.verbal.frases.simultaneo.recuerdo.verdaderofalso | 131 |
| braindamage.atencio.selectiva.figurassuperpuestas | 132 |
| braindamage.memoria.verbal.assocparpalb.pressimul.reconeixm.selecciona | 132 |
| braindamage.memoria.verbal.frases.secuencial.recuerdo.preguntas | 141 |
| braindamage.memoria.visual.associmgpalb.simultanea.recuerdo.mateixordre | 143 |
| braindamage.memoria.visual.associmgpalb.secuencial.reconocimiento.decideix | 157 |
| braindamage.memoria.verbal.frases.simultaneo.recuerdo.preguntas | 165 |
| braindamage.calculo.mental.vamosacomprar | 167 |
| braindamage.calculo.mental.problemasaritmeticos | 189 |
| braindamage.memoria.verbal.frases.simultaneo.reconocimiento.pregtest | 190 |
| braindamage.memoria.visual.associmgpalb.simultanea.reconocimiento.selecciona | 204 |
| braindamage.memoria.visual.associmgpalb.simultanea.reconocimiento.decideix | 213 |
| braindamage.funcionsexecutives.categoritzacio.agruparparaules | 220 |
| braindamage.memoria.verbal.apreserpalb.pressimul.reconeixm.selecciona | 223 |
| braindamage.memoria.verbal.assocparpalb.pressimul.reconeixm.relaciona | 225 |
| braindamage.memoria.verbal.frases.secuencial.reconocimiento.pregtest | 226 |
| braindamage.calculo.mental.series | 234 |
| braindamage.memoria.verbal.assocparpalb.presseque.reconeixm.relaciona | 238 |
| braindamage.orientacion.temporoespacial.mirarelreloj | 238 |
| braindamage.orientacion.temporoespacial.orientaciontemporal | 241 |
| braindamage.memoria.visual.associmgpalb.secuencial.reconocimiento.selecciona | 242 |
| braindamage.memoria.verbal.apreserpalb.presseque.reconeixm.decideix | 246 |
| braindamage.calculo.mental.calculomental | 250 |
| braindamage.atencio.selectiva.diferencies | 255 |
| braindamage.memoria.treball.dibuixosreconeixement | 255 |
| braindamage.orientacion.temporoespacial.viajando | 262 |
| braindamage.calculo.mental.euros | 271 |
| braindamage.gnosias.visuales.esquemacorporal | 272 |
| braindamage.gnosias.visuales.objetoscotidianos | 277 |
| braindamage.memoria.visual.escenes.record | 280 |
| braindamage.memoria.verbal.recuerdoserienumeros.reconocimientosecuencialserienumeros | 283 |
| braindamage.calculo.mental.sudoku | 286 |
| braindamage.gnosias.visuales.emociones | 288 |
| braindamage.calculo.mental.ordenaciondenumeros | 298 |
| braindamage.memoria.visual.associmgpalb.secuencial.reconocimiento.relaciona | 302 |
| braindamage.gnosias.visuales.colores | 303 |
| braindamage.memoria.verbal.recuerdoserienumeros.recuerdosimultaneoserienumeros | 307 |
| braindamage.atencio.sostinguda.mateixadirrecio | 312 |
| braindamage.gnosias.visuales.lugaresdelmundo | 315 |
| braindamage.memoria.visual.associmgpalb.ordretemp | 316 |
| braindamage.memoria.visual.escenes.reconeixement | 317 |
| braindamage.memoria.visual.associmgpalb.simultanea.reconocimiento.relaciona | 324 |
| braindamage.gnosias.visuales.escenas | 340 |
| braindamage.memoria.verbal.apreserpalb.presseque.reconeixm.selecciona | 358 |
| braindamage.atencio.selectiva.puzzle.competicio | 363 |
| braindamage.memoria.verbal.recuerdoserienumeros.reconocimientosimultaneoserienumeros | 368 |
| braindamage.atencio.sostinguda.difdirec | 369 |
| braindamage.memoria.verbal.recuerdoserienumeros.recuerdosecuencialserienumeros | 374 |
| braindamage.funcionsexecutives.inhibicio.gonogoposicio | 378 |
| braindamage.atencio.dividida.laberintmatematic.exercici | 383 |
| braindamage.funcionsexecutives.categoritzacio.paraulesmateixacategoria | 386 |
| braindamage.funcionsexecutives.inhibicio.gonogoest | 394 |
| braindamage.funcionsexecutives.flexibilitat.platforms | 442 |
| braindamage.atencio.dividida.laberintmatematic.competicio | 447 |
| braindamage.memoria.verbal.text.reconeixement.reconpregunta | 450 |
| braindamage.funcionsexecutives.planificacio.enfonsarlaflota | 464 |
| braindamage.atencio.dividida.operconc | 515 |
| braindamage.memoria.verbal.frases.secuencial.reconocimiento.ordena | 521 |
| braindamage.atencio.selectiva.puzzle.exercici | 526 |
| braindamage.memoria.verbal.frases.simultaneo.reconocimiento.ordena | 536 |
| braindamage.memoria.visual.dibuixos.dibuixosordretemporal | 546 |
| braindamage.atencio.selectiva.aparellament | 553 |
| braindamage.atencio.selectiva.soupletters | 568 |
| braindamage.atencio.sostinguda.liniarecta | 590 |
| braindamage.memoria.verbal.assocparpalb.ordretemp | 624 |
| braindamage.funcionsexecutives.sequenciacio.serie | 668 |
| braindamage.atencio.selectiva.bossamonedes | 677 |
| braindamage.memoria.visual.simon | 686 |
| braindamage.atencio.dividida.submari | 693 |
| braindamage.funcionsexecutives.sequenciacio.fragments | 707 |
| braindamage.funcionsexecutives.sequenciacio.serieciclica | 724 |
| braindamage.memoria.verbal.text.reconeixement.reconordena | 753 |
| braindamage.atencio.dividida.globallocal | 763 |
| braindamage.funcionsexecutives.flexibilitat.cercle | 775 |
| braindamage.funcionsexecutives.categoritzacio.categoritzacio3 | 788 |
| braindamage.funcionsexecutives.planificacio.penjat | 805 |
| braindamage.funcionsexecutives.planificacio.quatro | 826 |
| braindamage.funcionsexecutives.flexibilitat.zigurat | 859 |
| braindamage.funcionsexecutives.planificacio.quatreratlla | 860 |
| braindamage.memoria.treball.mateixacategoria | 865 |
| braindamage.funcionsexecutives.categoritzacio.categoritzacio2 | 882 |
| braindamage.funcionsexecutives.inhibicio.gonogojoc | 889 |
| braindamage.funcionsexecutives.sequenciacio.construiroracio | 889 |
| braindamage.atencio.sostinguda.bingo | 916 |
| braindamage.memoria.treball.simultani | 931 |
| braindamage.funcionsexecutives.planificacio.trencaclosques | 989 |
| braindamage.memoria.visual.memory | 1006 |
| braindamage.memoria.treball.ordretemporal | 1012 |
| braindamage.memoria.treball.sequencial | 1018 |
| braindamage.memoria.treball.posicio | 1062 |
| braindamage.funcionsexecutives.planificacio.laberint | 1116 |
| **Total of executions** | **44,814** |

Table S2. More frequently executed tasks

| **Task identifier** | **number of executions** |
| --- | --- |
| braindamage.atencio.selectiva.diferencies | 255 |
| braindamage.memoria.treball.dibuixosreconeixement | 255 |
| braindamage.orientacion.temporoespacial.viajando | 262 |
| braindamage.calculo.mental.euros | 271 |
| braindamage.gnosias.visuales.esquemacorporal | 272 |
| braindamage.gnosias.visuales.objetoscotidianos | 277 |
| braindamage.memoria.visual.escenes.record | 280 |
| braindamage.memoria.verbal.recuerdoserienumeros.reconocimientosecuencialserienumeros | 283 |
| braindamage.calculo.mental.sudoku | 286 |
| braindamage.gnosias.visuales.emociones | 288 |
| braindamage.calculo.mental.ordenaciondenumeros | 298 |
| braindamage.memoria.visual.associmgpalb.secuencial.reconocimiento.relaciona | 302 |
| braindamage.gnosias.visuales.colores | 303 |
| braindamage.memoria.verbal.recuerdoserienumeros.recuerdosimultaneoserienumeros | 307 |
| braindamage.atencio.sostinguda.mateixadirrecio | 312 |
| braindamage.gnosias.visuales.lugaresdelmundo | 315 |
| braindamage.memoria.visual.associmgpalb.ordretemp | 316 |
| braindamage.memoria.visual.escenes.reconeixement | 317 |
| braindamage.memoria.visual.associmgpalb.simultanea.reconocimiento.relaciona | 324 |
| braindamage.gnosias.visuales.escenas | 340 |
| braindamage.memoria.verbal.apreserpalb.presseque.reconeixm.selecciona | 358 |
| braindamage.atencio.selectiva.puzzle.competicio | 363 |
| braindamage.memoria.verbal.recuerdoserienumeros.reconocimientosimultaneoserienumeros | 368 |
| braindamage.atencio.sostinguda.difdirec | 369 |
| braindamage.memoria.verbal.recuerdoserienumeros.recuerdosecuencialserienumeros | 374 |
| braindamage.funcionsexecutives.inhibicio.gonogoposicio | 378 |
| braindamage.atencio.dividida.laberintmatematic.exercici | 383 |
| braindamage.funcionsexecutives.categoritzacio.paraulesmateixacategoria | 386 |
| braindamage.funcionsexecutives.inhibicio.gonogoest | 394 |
| braindamage.funcionsexecutives.flexibilitat.platforms | 442 |
| braindamage.atencio.dividida.laberintmatematic.competicio | 447 |
| braindamage.memoria.verbal.text.reconeixement.reconpregunta | 450 |
| braindamage.funcionsexecutives.planificacio.enfonsarlaflota | 464 |
| braindamage.atencio.dividida.operconc | 515 |
| braindamage.memoria.verbal.frases.secuencial.reconocimiento.ordena | 521 |
| braindamage.atencio.selectiva.puzzle.exercici | 526 |
| braindamage.memoria.verbal.frases.simultaneo.reconocimiento.ordena | 536 |
| braindamage.memoria.visual.dibuixos.dibuixosordretemporal | 546 |
| braindamage.atencio.selectiva.aparellament | 553 |
| braindamage.atencio.selectiva.soupletters | 568 |
| braindamage.atencio.sostinguda.liniarecta | 590 |
| braindamage.memoria.verbal.assocparpalb.ordretemp | 624 |
| braindamage.funcionsexecutives.sequenciacio.serie | 668 |
| braindamage.atencio.selectiva.bossamonedes | 677 |
| braindamage.memoria.visual.simon | 686 |
| braindamage.atencio.dividida.submari | 693 |
| braindamage.funcionsexecutives.sequenciacio.fragments | 707 |
| braindamage.funcionsexecutives.sequenciacio.serieciclica | 724 |
| braindamage.memoria.verbal.text.reconeixement.reconordena | 753 |
| braindamage.atencio.dividida.globallocal | 763 |
| braindamage.funcionsexecutives.flexibilitat.cercle | 775 |
| braindamage.funcionsexecutives.categoritzacio.categoritzacio3 | 788 |
| braindamage.funcionsexecutives.planificacio.penjat | 805 |
| braindamage.funcionsexecutives.planificacio.quatro | 826 |
| braindamage.funcionsexecutives.flexibilitat.zigurat | 859 |
| braindamage.funcionsexecutives.planificacio.quatreratlla | 860 |
| braindamage.memoria.treball.mateixacategoria | 865 |
| braindamage.funcionsexecutives.categoritzacio.categoritzacio2 | 882 |
| braindamage.funcionsexecutives.inhibicio.gonogojoc | 889 |
| braindamage.funcionsexecutives.sequenciacio.construiroracio | 889 |
| braindamage.atencio.sostinguda.bingo | 916 |
| braindamage.memoria.treball.simultani | 931 |
| braindamage.funcionsexecutives.planificacio.trencaclosques | 989 |
| braindamage.memoria.visual.memory | 1006 |
| braindamage.memoria.treball.ordretemporal | 1012 |
| braindamage.memoria.treball.sequencial | 1018 |
| braindamage.memoria.treball.posicio | 1062 |
| braindamage.funcionsexecutives.planificacio.laberint | 1116 |
| **Total number of executions** | **38,177** |

Table S3. Spearman’s correlations for RAVLT (admission and discharge) DIGITS (admission and discharge) and Age at admission

|  | Age | Digits-Adm | RAVLT75−Adm | RAVLT15−Adm | RAVLT15R−Adm | DIGITS−Dis | RAVLT75−Dis | RAVLT15−Dis | RAVLT15R−Dis |
| --- | --- | --- | --- | --- | --- | --- | --- | --- | --- |
| Age | 1 |  |  |  |  |  |  |  |  |
| Digits-Adm | ns | 1 |  |  |  |  |  |  |  |
| RAVLT075−Adm | -0.18 | 0.32 | 1 |  |  |  |  |  |  |
| RAVLT015−Adm | -0.17 | 0.26 | **0.78** | 1 |  |  |  |  |  |
| RAVLT015R−Adm | -0.21 | 0.20 | **0.64** | **0.68** | 1 |  |  |  |  |
| DIGITS−Dis | ns | **0.64** | 0.27 | 0.20 | 0.13^a^ | 1 |  |  |  |
| RAVLT075−Dis | -0.22 | 0.30 | **0.70** | **0.67** | **0.54** | 0.34 | 1 |  |  |
| RAVLT015−Dis | -0.22 | 0.20 | **0.65** | **0.73** | **0.61** | 0.20 | **0.81** | 1 |  |
| RAVLT015R−Dis | -0.24 | 0.23 | **0.54** | **0.59** | **0.67** | 0.26 | **0.69** | **0.69** | 1 |

^a^ P < 0.05; P < 0.01 otherwise; ns: non-significant; RAVLT: Rey Auditory Verbal Learning Test; Adm: Admission; Dis: Discharge
